# Supplementary material for: Natural History of Stargardt Disease: The Longest Follow-Up Cohort Study
Source: Genes (Basel). 2023 Jul 2;14(7):1394. doi: 10.3390/genes14071394 (PMC10379489; doi:10.3390/genes14071394)
Supplement: Supplementary file 1 [file genes-14-01394-s001.zip › genes-2473122-supplementary/Supplementary Figure S1.pdf]

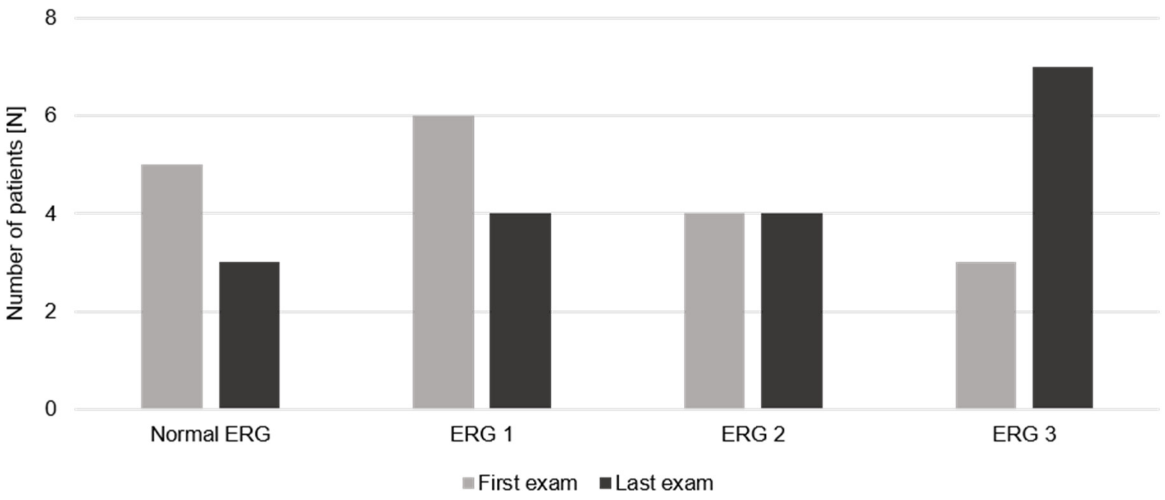

Figure S1. Representation of ERG groups for the first and last exams. According to the ERG groups, at the beginning of the follow-up, 5 patients showed no ERG abnormalities, whereas 6 patients were classified as ERG group 1, 4 as group 2, and 3 as group 3. At the end of the follow-up, 4 patients were classified as ERG group 1, 4 as group 2, and 7 as group 3, whereas 3 patients showed no ERG abnormalities. For the course of follow-up, 8/18 (44%) of the patients showed ERG progression: 6 patients progressed for one group and 2 patients for two groups.
