# Supplementary material for: Natural History of Stargardt Disease: The Longest Follow-Up Cohort Study
Source: Genes (Basel). 2023 Jul 2;14(7):1394. doi: 10.3390/genes14071394 (PMC10379489; doi:10.3390/genes14071394)
Supplement: Supplementary file 1 [file genes-14-01394-s001.zip › genes-2473122-supplementary/Supplementary Figure S2.pdf]

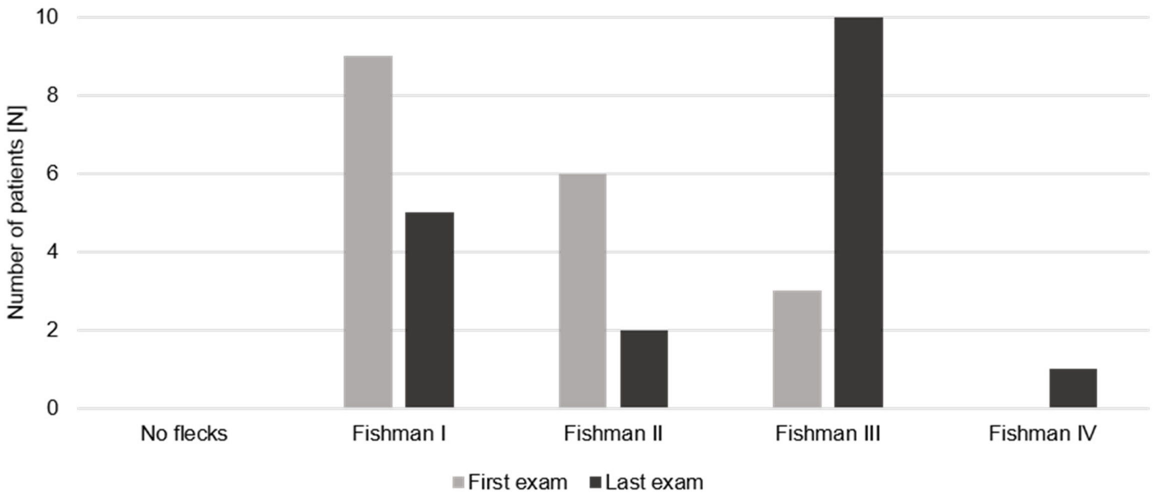

Figure S2. Representation of Fishman stages for the first and last exams. According to the Fishman classification, at the beginning of the follow-up, 9 patients were classified as stage I, 6 patients as stage II and 3 patients as stage III. None of the patients had a normal fundus appearance. Moreover, none of them was in Fishman stage IV. At the end of the follow-up, 5 patients were classified as Fishman stage I, 2 as stage II, 10 as stage III, whereas 1 patient was in Fishman stage IV. Fundus appearance stayed stable in 8/18 (44%) patients, while it progressed in 10/18 (56%) patients. In 7 patients, it progressed for one Fishman stage and in 3 patients for two Fishman stages.
