# Supplementary material for: Natural History of Stargardt Disease: The Longest Follow-Up Cohort Study
Source: Genes (Basel). 2023 Jul 2;14(7):1394. doi: 10.3390/genes14071394 (PMC10379489; doi:10.3390/genes14071394)
Supplement: Supplementary file 1 [file genes-14-01394-s001.zip › genes-2473122-supplementary/Supplementary Table S1.pdf]

Table S1. Clinical characteristics

P	Sex	*ABCA4* variant 1	*ABCA4* variant 2	Age at onset [years]	Follow-up period [years]	Duration between onset and the first exam [years]	Age at the first and last exams [years]	VA at the first and last exams [Snellen]	VA loss per year [Snellen]	DDAF area at the first and last exams [mm^2]	Increase of DDAF area per year [mm^2]	ERG group at the first and last exams	Fishman stage at the first and last exams
1*	F	c.4234C>T; p.(Gln1412*)	c.5882G>A; p.(Gly1961Glu)	46	26	0	46	0.8	0.025	0.47	0.296	normal ERG	II
							72	0.16		8.18		1	III
2	F	c.2041C>T; p.(Arg681*)	c.5413A>G; p.(Asn1805Asp)	15	25	14	29	0.1	0.002	13.70	2.596	3	III
							54	0.05		78.61		3	IV
3*	M	c.5882G>A; p.(Gly1961Glu)	c.[5461-10T>C;5603A>T]; p.[Thr1821Aspfs*6,Thr1821Valfs*13;(Asn1868Ile)]	7	13	15	22	0.2	0.004	0.02	0.141	1	I
							35	0.15		1.85		3	I
4*	F	c.1648G>A; p.(Gly550Arg)	c.5882G>A; p.(Gly1961Glu)	14	21	30	44	0.2	0.009	0.20	0.011	normal ERG	I
							65	0.02		0.43		normal ERG	I
5	F	c.2041C>T; p.(Arg681*)	c.6089G>A; p.(Arg2030Gln)	15	23	4	19	0.15	0.005	1.43	0.707	1	I
							42	0.03		17.69		2	III
6	F	c.5175dup(;)5603A>T; p.(Thr1726Aspfs*61)(;)(Asn1868Ile)	c.5714+5G>A; p.[=,Glu1863Leufs*33]	18	11	9	27	0.4	0.034	11.60	0.929	2	III
							38	0.03		21.82		2	III
7	F	c.5175dup(;)5603A>T; p.(Thr1726Aspfs*61)(;)(Asn1868Ile)	c.5714+5G>A; p.[=,Glu1863Leufs*33]	25	11	6	31	0.8	0.071	11.18	4.361	2	III
							42	0.02		59.14		3	III
8	M	c.688T>A; p.(Cys230Ser)	c.4539+2T>C; p.(?)	11	19	2	13	0.3	0.015	0.24	0.504	2	I
							32	0.03		9.81		2	III
9*	M	c.2041C>T; p.(Arg681*)	c.5603A>T; p.(Asn1868Ile)	30	17	0	30	0.8	0.035	0.12	0.050	normal ERG	I
							47	0.2		0.96		1	II
10*	F	c.1918C>G(;)5603A>T; p.(Pro640Ala)(;)(Asn1868Ile)	c.5882G>A; p.(Gly1961Glu)	38	20	1	39	0.8	0.030	0.30	0.021	1	I
							59	0.2		0.71		1	I
11*	F	c.5175dup(;)5603A>T; p.(Thr1726Aspfs*61)(;)(Asn1868Ile)	c.5882G>A; p.(Gly1961Glu)	19	20	6	25	0.2	-0.005	0.00	0.002	normal ERG	I
							45	0.3		0.03		normal ERG	I

12*	F	c.4919G>A; p.(Arg1640Gln)	c.5603A>T; p.(Asn1868Ile)	20	26	1	21	0.6	0.022	2.43	0.269	1	I
							47	0.03		9.43		2	III
13*	F	c.2041C>T; p.(Arg681*)	c.5603A>T; p.(Asn1868Ile)	20	16	0	20	0.2	0.003	0.90	0.083	1	I
							36	0.16		2.23		1	I
14	F	c.6445C>T; p.(Arg2149*)	c.6445C>T; p.(Arg2149*)	7	10	0	7	0.1	0.008	0.03	1.152	3	II
							17	0.02		11.54		3	II
15	F	c.6445C>T; p.(Arg2149*)	c.6445C>T; p.(Arg2149*)	9	12	1	10	0.3	0.024	0.91	1.574	2	II
							22	0.02		19.80		3	III
16	M	c.5977del; p.(Ser1993fs)	c.5461-10T>C; p.[Thr1821Aspfs*6,Thr1821Valfs*13]	8	11	1	9	0.1	0.005	3.81	1.129	3	II
							20	0.04		16.23		3	III
17	M	c.[5461-10T>C;5603A>T]; p.[Thr1821Aspfs*6,Thr1821Valfs*13;(Asn1868Ile)]	c.5714+5G>A; p.[=,Glu1863Leufs*33]	11	18	5	16	0.2	0.007	0.16	0.1628	1	II
							34	0.03		3.09		3	III
18	F	c.1570A>G; p.(Lys524Glu)	c.5714+5G>A; p.[=,Glu1863Leufs*33]	17	11	1	18	0.3	0.009	0.15	0.4109	normal ERG	II
							29	0.2		4.67		normal ERG	III

Patients 6 and 7, as well as 14 and 15 are siblings. Eight patients with p.(Gly1961Glu) or p.(Asn1868Ile) allele are marked with a star. Abbreviation explanation: F—female, M—male.
